# Supplementary material for: Inhibitory Effect of Zinc on Colorectal Cancer by Granzyme B Transcriptional Regulation in Cytotoxic T Cells
Source: Int J Mol Sci. 2023 May 29;24(11):9457. doi: 10.3390/ijms24119457 (PMC10253514; doi:10.3390/ijms24119457)
Supplement: Supplementary file 1 [file ijms-24-09457-s001.zip › ijms-2393471-supplementary/ijms-2393471-supplementary.pdf]

Supplemental Figures

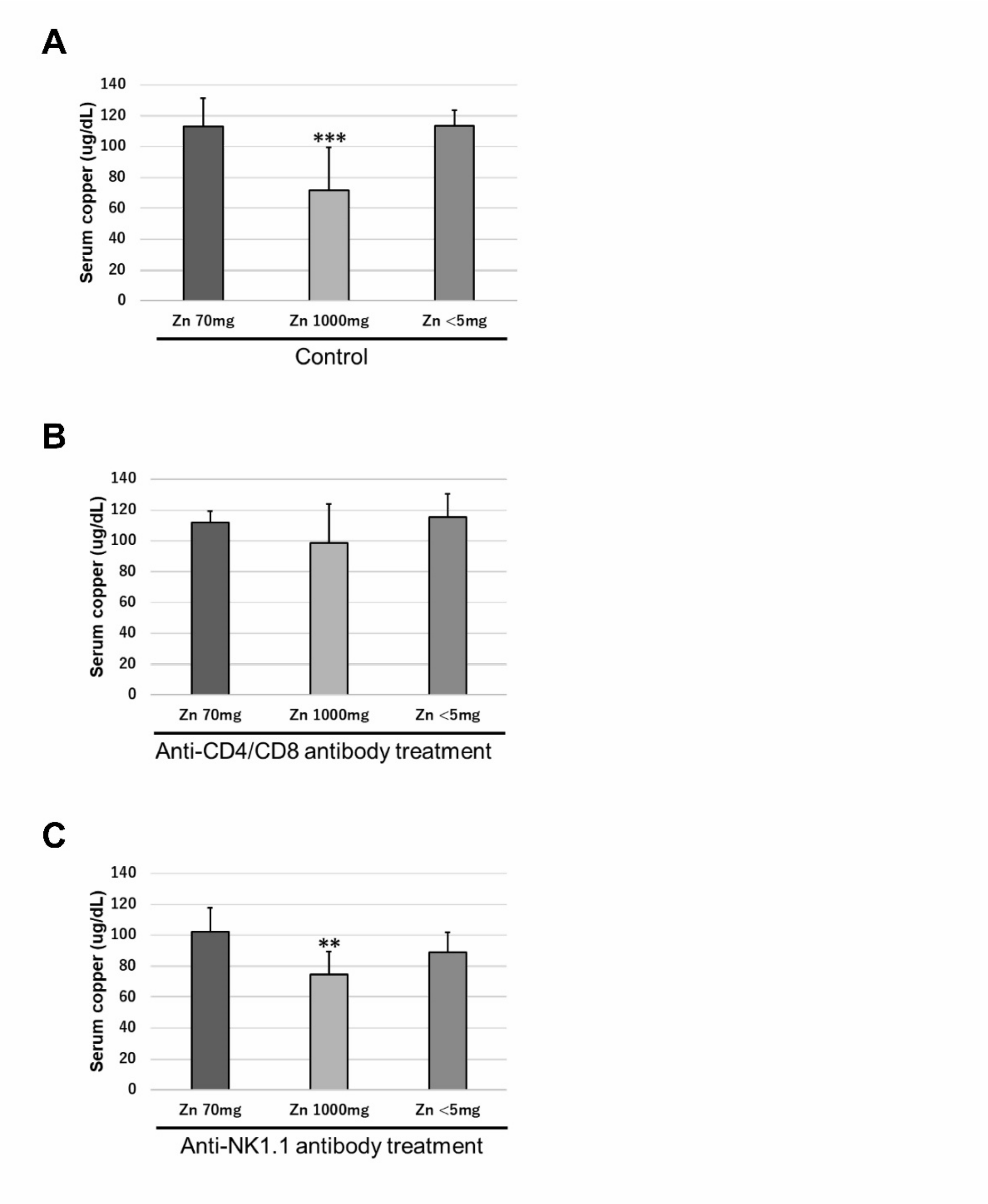

Figure S1. Effect of zinc administration on serum copper levels.

Copper levels in serum of ICR mice collected at week 20 were measured. (A) serum copper levels in control mice (Zn=70mg n=29, Zn=1000mg n=23, Zn<5mg n=17); (B) serum copper levels in anti-CD4/CD8 antibodies administered mice (Zn=70mg n=6, Zn=1000mg n=7, Zn<5mg n=9); and (C) serum copper levels in

anti-NK1.1 antibody administered mice (Zn=70mg n=7, Zn=1000mg n=8, Zn<5mg n=9). (bar: SD; **p<0.01; ***p<0.001; Tukey's HSD test)

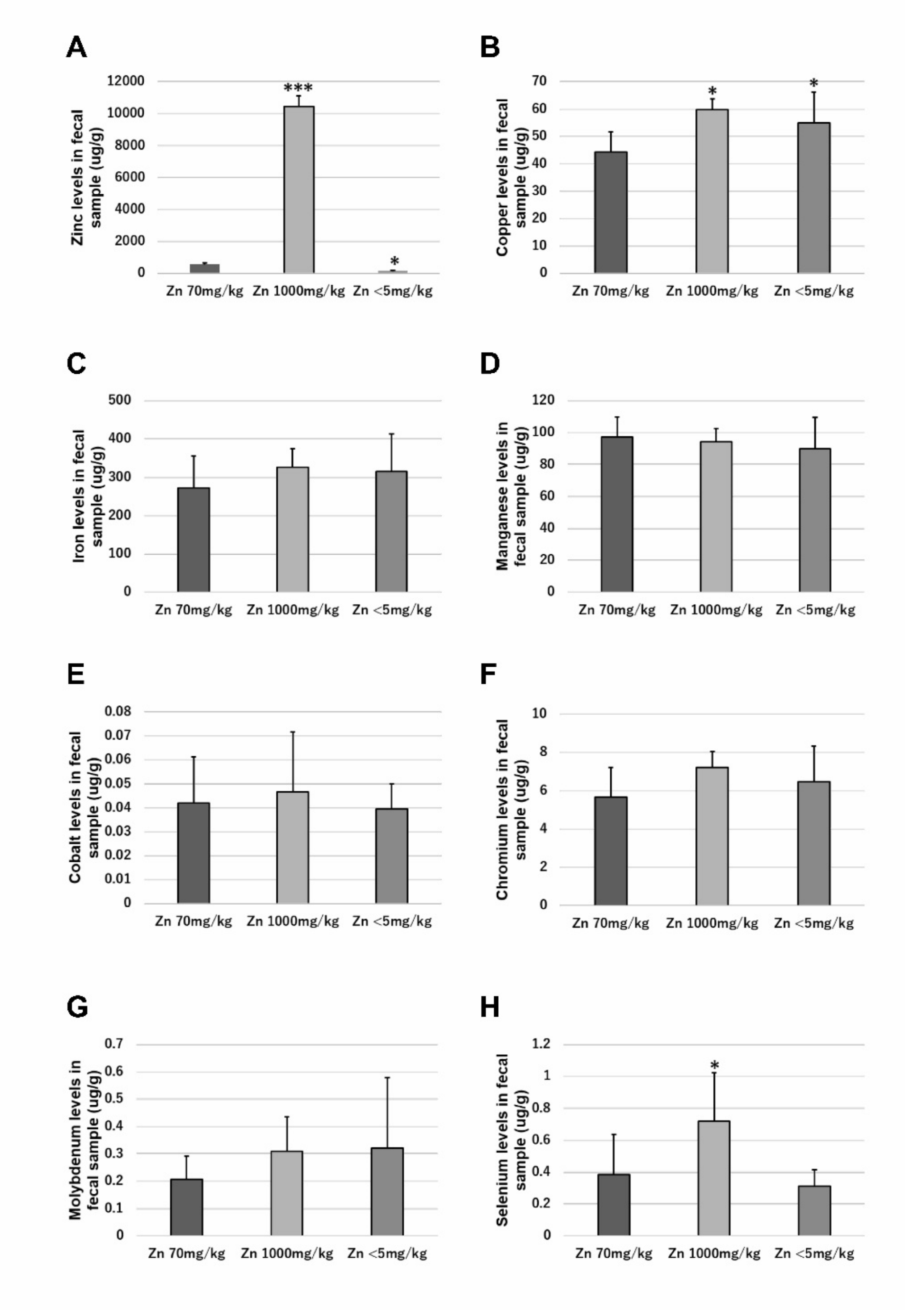

Figure S2. Effect of zinc administration on trace metals in mouse feces.

Trace metals in feces collected from ICR mice (Zn=70mg n=9, Zn=1000mg n=5,

Zn<5mg n=9) at week 20 of the experiment were measured by ICP-MS. (A) zinc levels in fecal sample; (B) copper levels in fecal sample; (C) iron levels in fecal sample; (D) manganese levels in fecal sample; (E) cobalt levels in fecal sample; (F) chromium levels in fecal sample; (G) molybdenum levels in fecal sample; and (H) selenium levels in fecal sample. (bar: SD; *p<0.05; ***p<0.001; Tukey's HSD test)
